# Randomised controlled trial of a digitally assisted low intensity intervention to promote personal recovery in persisting psychosis: SMART-Therapy study protocol

**DOI:** 10.1186/s12888-016-1024-1

**Published:** 2016-09-07

**Authors:** Neil Thomas, John Farhall, Fiona Foley, Susan L. Rossell, David Castle, Emma Ladd, Denny Meyer, Cathrine Mihalopoulos, Nuwan Leitan, Cassy Nunan, Rosalie Frankish, Tara Smark, Sue Farnan, Bronte McLeod, Leon Sterling, Greg Murray, Ellie Fossey, Lisa Brophy, Michael Kyrios

**Affiliations:** 1 Centre for Mental Health, Swinburne University of Technology, PO Box 218, Hawthorn, VIC 3122 Australia; 2 Monash Alfred Psychiatry Research Centre, Monash University and The Alfred, Melbourne, VIC 3004 Australia; 3 Department of Psychology and Counselling, La Trobe University, Melbourne, VIC 3086 Australia; 4 NorthWestern Mental Health, Royal Melbourne Hospital, Melbourne, VIC 3050 Australia; 5 Department of Psychiatry, St Vincent’s Hospital, Fitzroy, VIC 3065 Australia; 6 Department of Psychiatry, University of Melbourne, Parkville, VIC 3052 Australia; 7 Wellways Australia, Fairfield, VIC 3068 Australia; 8 Centre for Population Health Research, Deakin University, Burwood, VIC 3125 Australia; 9 Centre for Design Innovation, Swinburne University of Technology, Hawthorn, VIC 3122 Australia; 10 Department of Computing and Information Systems, University of Melbourne, Parkville, VIC 3052 Australia; 11 Department of Occupational Therapy, Monash University - Peninsula Campus, Frankston, VIC 3199 Australia; 12 Living with Disability Research Centre, La Trobe University, Melbourne, VIC 3086 Australia; 13 Mind Australia, Heidelberg, VIC 3084 Australia; 14 Melbourne School of Population and Global Health, University of Melbourne, Parkville, VIC 3052 Australia; 15 Australian National University, Canberra, ACT 2601 Australia

**Keywords:** Randomised controlled trial (RCT), Psychosis, Schizophrenia, Digital health, e-therapy, Tablet computers, Peer support, Illness self-management, Personal recovery

## Abstract

**Background:**

Psychosocial interventions have an important role in promoting recovery in people with persisting psychotic disorders such as schizophrenia. Readily available, digital technology provides a means of developing therapeutic resources for use together by practitioners and mental health service users. As part of the Self-Management and Recovery Technology (SMART) research program, we have developed an online resource providing materials on illness self-management and personal recovery based on the Connectedness-Hope-Identity-Meaning-Empowerment (CHIME) framework. Content is communicated using videos featuring persons with lived experience of psychosis discussing how they have navigated issues in their own recovery. This was developed to be suitable for use on a tablet computer during sessions with a mental health worker to promote discussion about recovery.

**Methods/Design:**

This is a rater-blinded randomised controlled trial comparing a low intensity recovery intervention of eight one-to-one face-to-face sessions with a mental health worker using the SMART website alongside routine care, versus an eight-session comparison condition, befriending. The recruitment target is 148 participants with a schizophrenia-related disorder or mood disorder with a history of psychosis, recruited from mental health services in Victoria, Australia. Following baseline assessment, participants are randomised to intervention, and complete follow up assessments at 3, 6 and 9 months post-baseline. The primary outcome is personal recovery measured using the Process of Recovery Questionnaire (QPR). Secondary outcomes include positive and negative symptoms assessed with the Positive and Negative Syndrome Scale, subjective experiences of psychosis, emotional symptoms, quality of life and resource use. Mechanisms of change via effects on self-stigma and self-efficacy will be examined.

**Discussion:**

This protocol describes a novel intervention which tests new therapeutic methods including in-session tablet computer use and video-based peer modelling. It also informs a possible low intensity intervention model potentially viable for delivery across the mental health workforce.

**Trial registration:**

NCT02474524, 24 May 2015, retrospectively registered during the recruitment phase.

## Background

In spite of early intervention and the routine use of antipsychotic medication, many people with psychotic disorders live with persisting symptoms and disability [1]. For this group, alongside good clinical care and treatment, interventions are needed to help people to self-manage and adjust to persisting symptoms, and to maximise quality of life. Psychotherapeutic interventions such as cognitive behavioural therapy for psychosis have been heavily recommended in clinical practice guidelines [2–4]. However, psychotherapies, which require lengthy therapist training, have proven hard to deliver on a widespread routine basis within mental health services [5–8], and are provided only to a small minority of consumers [9]. In addressing need, there may be value in developing interventions that make use of structured therapeutic resources that support practitioners in their delivery, and which can be delivered with a combination of in-session and self-guided use. These interventions, often referred to as *low intensity interventions* [10], are amenable to delivery without extensive specific training or prerequisite skill, and have the potential to be delivered by a broad mental health workforce, allowing for greater integration into routine service provision.

Group/classroom-format illness self-management courses accompanied by workbooks have already shown promise [11–15]. In parallel, online digital tools are beginning to be developed for psychosis [16–18]. Integrating digital tools into face-to-face mental health practice has significant implementation potential. The blending of face-to-face and online service delivery allows for practitioner support in using a digital tool, promotes more in-depth and individualised reflection upon standardised content, and helps to sustain engagement with materials. Strengths of the digital format include being easily disseminated, being directly accessible by consumers, offering more interactive therapeutic materials than workbooks, allowing for mobile device use to support self-management throughout the day, and potentially allowing peer-to-peer interaction to promote the development of supportive peer communities [16, 18].

Although initially having much poorer access to the Internet than the broader population [19], the majority of consumers with persisting psychosis are now online, particularly via internet-enabled smartphones [20, 21], and consumers have expressed positive attitudes towards use of digital technology in mental health care [22]. Initial trials of digital interventions have shown self-guided use to be feasible [18]. Similarly, workers within mental health services welcome the in-session use of digital technology as part of their work with this population [23]. To test the integration of digital technology into routine mental health interactions, we sought to develop and trial a manualised therapeutic intervention that blended face-to-face interaction with a digital tool, and that could be used by mental health workers without an advanced level of training.

In developing such an intervention, a key therapeutic target is *personal recovery*, a domain which is now prioritised in mental health services worldwide [24–30]. Personal recovery is often contrasted with the traditional treatment targets of *clinical recovery* (minimisation of symptoms), and *functional recovery* (improving social functioning), as a process of living a full life irrespective of ongoing symptoms or disability [30–32]. Anthony [33] defines personal recovery as “a deeply personal, unique process of changing one's attitudes, values, feelings, goals, skills and/or roles. It is a way of living a satisfying, hopeful, and contributing life even with limitations caused by the illness” (p. 527). This conceptualisation of recovery has been strongly influenced by consumer accounts of their own recovery [30, 32]. Although recovery is typically characterised as an individual journey, synthesis of mental health service consumer accounts has led to consensus around particular processes as important in promoting personal recovery: becoming empowered and taking responsibility for self-managing mental health; developing hope and optimism; establishing a self-identity accepting of but not defined by illness; developing new meaning and life goals; and connecting with others [34–36].

How mental health services can most effectively support these recovery processes is little researched as yet. Although staff training in recovery-oriented practice is a common model, a major trial of training in recovery-oriented practice observed limited impact on a measure of these recovery processes [37]. On the other hand, positive outcomes for measures of recovery have been found for group-format self-management interventions that incorporate material on recovery [11–13]. In the most widely used interventions, lived experience of mental health problems plays a key role in the intervention, with the intervention commonly facilitated by a peer who is in recovery, and with the group format being used to elicit discussion among peers [38]. This accords with a recovery literature highlighting peer contact particularly important in promoting recovery from psychosis [32]. Specifically, qualitative studies suggest peers providing positive role models, peers inspiring hope and motivation, and peer-to-peer interaction promoting social connection are core to the benefits of peer involvement [39].

Use of a digital medium provides opportunities to bring peer lived experience into the context of face-to-face mental health interactions with non-peer mental health practitioners. Audiovisual media can be utilised to feature peers as the main communicators of content, consistent with the origins of the concept of personal recovery in consumer narratives. Doing so potentially supports the benefits of peer positive role modelling and hope [40], and portraying peers discussing their recovery, in a humanised and dignified way, may be empowering and challenging of negative stereotypes about mental illness [41]. In addition, the Internet can be used to support people using the tool to communicate with each other, potentially allowing for contribution of personal content, and the formation of supportive peer communities [42].

## Methods/Design

### Aims

This study (acronym SMART-Therapy) is part of a broader research program (Self-Management and Recovery Technology) developing and trialling digital resources for specialist mental health services. It is a randomised controlled trial of a discrete psychosocial intervention to promote personal recovery in people with persisting psychosis. The intervention adopts a low intensity delivery model blending a course of individual face-to-face sessions with use of a website featuring lived experience content on recovery. The primary objective is to determine if this intervention will be superior to a time-equivalent comparison intervention that incorporates exploration of non-recovery-related digital resources on a measure of consumer-defined personal recovery. We will also examine a number of secondary outcomes, interview people about their experience of the intervention, use data as part of a broader economic evaluation, and examine mechanisms of change.

In examining the mechanisms of the intervention, we hypothesise effects on personal recovery via two measurable processes: (a) mental illness self-stigma and (b) self-efficacy for mental health recovery. Self-stigma, also referred to as internalised stigma, refers to the extent to which an individual adopts negative community stereotypes about mental illness and the extent to which these exert an influence on experience and behaviour [43]. For persons with persisting psychosis, these may include stereotypes of being fundamentally different from others, of inherent dangerousness, of pessimistic long-term outcomes, and of being insufficiently capable of fulfilling social roles such as parenting and work [44]. Internalised stigma includes components such as negative stereotype endorsement, alienation and social withdrawal [45]. In studies of public stigma, contact with persons with mental illness that undermines negative stereotypes and humanises persons with a mental illness appears effective in reducing negative stereotypes [46]. We hypothesise that positive portrayal of peers may have a parallel effect on internalised stigma [41].

A concept derived from social learning theory, self-efficacy refers to the expectancies that an individual has that they will be able to successfully engage in a target behaviour [47]. Self-efficacy is a robust predictor of health behaviour change [48]. In line with social learning theory we predict that observing peers discussing engagement in recovery oriented behaviours will facilitate behaviour change in our participants via increased expectancies of being able to engage in similar behaviours.

### Design

The SMART-Therapy trial is designed as a randomised, controlled, assessor-blinded superiority trial, with two parallel groups, using a 1:1 allocation ratio. The two groups both receive eight sessions with a mental health worker in addition to their routine care during a three month window, with one group being delivered the recovery intervention, and the other being delivered a befriending intervention as a comparison condition. Outcome measures are completed at baseline (prior to randomisation) and are repeated at 3, 6 and 9 months following baseline.

### Participants

The study is being conducted across multiple public community mental health services in the state of Victoria, Australia, including clinical mental health services, and not-for-profit organisation-run community mental health support services. Potential participants are identified via referral from practitioners in collaborating services, supplemented by review of case-lists, plus advertising within services, and in print and online social media.

Inclusion criteria are: (a) age between 18 and 65 years inclusive; (b) diagnosis of a nonorganic psychotic disorder (schizophrenia-related disorder or bipolar disorder or major depressive disorder with psychotic features present within the past 2 years), confirmed using the Structured Clinical Interview for DSM-IV-TR Axis I Disorders (SCID) [49]; (c) sufficient conversational English for meaningful participation; (d) overall intellectual functioning within normal limits (having an IQ greater than 70, as estimated by the Wechsler Test of Adult Reading (WTAR) [50]) to ensure they have the cognitive capacity to provide consent, in addition to being able to adequately engage with the intervention. Exclusion criteria are: (e) initiation of a new antipsychotic medication, or commencement or completion of a formal psychological treatment, within the previous 8 weeks; (f) inpatient admission within the previous 8 weeks.

The recruitment target is 148 participants, providing 80 % power to detect medium effect sizes (*d* = 0.5) at α = .05 with 15 % attrition. This was based on an average effect size of *d* = 0.53 observed in a meta-analysis of online interventions [51].

### Interventions

Both conditions involve delivery of eight face-to-face one-to-one sessions of 50 min duration. Interventions are delivered by mental health support workers who are seconded to the project from roles in the not-for-profit community mental health support services sector. Workers in this sector were chosen to ensure that the intervention is suitable for delivery by the whole mental health workforce, rather than being restricted to postgraduate trained health professionals. These mental health workers are required to have a certificate level of training in mental health and at least 2 years of experience. They facilitate sessions for both conditions. Sessions are held alongside the participant’s usual routine treatment and care, which continues to be provided by their existing treating team without restriction from participation in the trial. Routine care would typically include antipsychotic medication, crisis support, symptom monitoring and case management, and may include receipt of formal psychological therapies.

### SMART

SMART sessions are structured around use of the SMART website on a tablet computer, which is used by the facilitator and participant together during sessions. The SMART website is Drupal-based, optimised for tablet computer or smartphone use, and accessible via any internet browser including home computer.

Content domains are primarily based on the Connectedness–Hope–Identity–Meaning–Empowerment (CHIME) framework, which has been derived as a synthesis of consumer accounts of key processes involved in recovery [35]. This is supplemented by basic symptom monitoring, coping enhancement, and behaviour change material from cognitive behavioural therapies. This creates seven self-management and recovery topics: (a) recovery, (b) managing stress, (c) physical health, (d) me, (e) empowerment, (f) relationships, and (g) life. Material for each topic is organized primarily around a series of videos, including a video introduction to the topic by a mental health consumer leader, followed by a combination of videos, text, and reflective exercises. The main videos are 2–3 min in length, each featuring a selection of persons with lived experience of psychosis reflecting on their experiences in relation to a particular aspect of recovery, and how they have navigated their mental health recovery in relation to this. Topics also include expert videos (a combination of peer leaders and non-peer academics) discussing conceptual points in more detail. Participants can share comments on each video with other users of the website in a comments feed. Due to the highly individual nature of recovery, and feedback during the consultation phase, resources were developed to include a pool of material that would be utilised in a flexible manner according to the participant’s own goals and recovery priorities. Participants’ entries for key exercises in each topic populate a “roadmap” page acting as a personalised summary of key points. Each topic also concludes by encouraging the person to identify key changes they intend to make, used to populate a task list. The site also contains a charting tool for self-monitoring stress, mood, sleep, physical health and self-esteem, and a peer-moderated member forum. Further details on the site development are detailed elsewhere [40].

Sessions involve use of the website to stimulate discussion about self-management and recovery. The worker has the following roles: (a) using website material to encourage the participant to reflect upon their own recovery, (b) facilitating the participant forming intentions to make changes on the basis of what has been discussed, (c) promoting familiarity with the site to independently use its features, and (d) encouraging the participant to use the site between sessions. The initial session includes orienting the participant to both the website and the concept of personal recovery. Participants are provided with their own login to the SMART website, and are introduced to the navigation, supported by a video on the site. This is followed by videos on the concept of personal recovery, and discussion about different material included in the website. This is used to identify priorities in the material for the therapist and participant to look at in subsequent sessions. In subsequent sessions, the worker and participant identify the area they want to focus on, with the core of the session involving a process of viewing website material together, using this as a prompt for discussion about the participant’s own recovery, and an invitation to complete reflective exercises on the site and/or make public posts about the content if willing. These sessions begin with a review of reflections since the last meeting, and any content which the person has used, and conclude by considering any changes the person would like to enact, and planning ongoing use of the site during the week.

### Befriending

Participants in the comparison condition receive sessions based upon a neutral social contact, involving shared activity and discussion about non-mental health topics such as current affairs, recent events and activities in the participant’s life, and the participant’s interests. This is a manualised intervention, the structure of which is based upon the befriending protocol of Bendall et al. [52], a credible and acceptable comparison condition [53], which has been used as a control condition to match for therapist time in a number of psychosocial intervention trials [54–56]. In this trial the standard protocol is augmented by use of the tablet computer to browse Internet sites related to participants’ interests, and as a potential means of facilitating discussion. This comparison condition was selected to provide a control for both therapist time, and for engagement in the use of technology.

In both groups, interventions are continued until, (a) all eight sessions are completed, (b) the end of the three month therapy delivery window is reached, or (c) the participant chooses to withdraw. If participants experience significant distress or deterioration in their mental state, researchers remind participants of their right to withdraw, and encourage them to make an informed choice about their ongoing participation. If participants wish to discontinue intervention sessions, they are still followed up for assessment if willing to do so.

Participant adherence to protocols will be monitored through records of session attendance. In secondary analyses, attendance of at least four sessions will be considered as the threshold for receiving a minimum dose of the intervention. Workers attend weekly supervision led by NT to ensure protocol fidelity. Worker fidelity will be further assessed by a researcher blind to participant allocation listening to a random sample of audio recordings of sessions, and being asked to allocate sessions to condition.

### Allocation and blinding

The trial uses minimisation randomisation, conducted using the software Minimpy (http://sourceforge.net/projects/minimpy). This generates random allocations based on the stratification parameters for each participant enrolled in the study. The randomisation procedure and setup of the Minimpy parameters was prepared by the study statistician to stratify by type of mental health service the person receives (clinical vs community sector vs both), symptom severity (Positive and Negative Syndrome Scale (PANSS) total ≥ vs < 67), and frequency of Internet use (daily vs less than daily). Following commencement of the trial, the software is operated by a person independent of the research team, who receives participant codes from the research team as each baseline assessment is completed, runs the allocation, and advises of allocations via return email.

Research staff conducting assessments are blind to participant allocation throughout the study. Participants are unavoidably aware of the conditions they have been randomised to. To reduce expectancy effects for superiority of the intervention over the control condition, the trial was framed as a comparison between two ways of integrating technology into mental health service delivery, with a *health* condition using technology to discuss mental health recovery, and a *social* condition using technology to support discussion about the person’s interests. To maintain assessor blinding, participants are regularly reminded not to divulge their allocated condition or any details of their intervention to assessment staff, as are their mental health workers. Breaches in blindness are recorded and addressed by changing the rater whenever possible. Blindness is also assessed at each assessment following the intervention by asking raters to nominate a guessed treatment condition for the participant and to indicate their level of confidence. The principal investigator (NT) and project manager (FF) are not blinded, and respond to any clinical and research issues during the trial that require knowledge of a participant’s’ condition.

### Measures

A schedule of assessments is provided in Table 1. Assessments are performed as close as possible to the allocated time-point. If the assessment cannot be performed within 6 weeks of the allocated time, this assessment is considered missed and the next assessment is performed at the allocated time.

**Table 1:** Participant timeline

	Study period					
	Enrolment	Allocation	Post-allocation			Close-out
Timepoint	Baseline		Week 1–12	3 months	6 months	9 months
	(*t*_0_)					
Enrolment						
Eligibility screen	X					
Informed consent	X					
Allocation		X				
Interventions						
SMART			X			
Befriending			X			
Assessments						
QPR	X			X	X	X
Schizophrenia Hope Scale	X			X	X	X
UCLA Loneliness Scale	X			X	X	X
PANSS	X			X	X	X
SEPS	X			X	X	X
DASS-21	X			X	X	X
AQoL8D	X			X	X	X
STAR-Patient	X			X	X	X
Therapy Evaluation				X		
Serious Adverse Events	X		X	X	X	X
RUQ	X			X	X	X
ISMI	X			X	X	X
Self-efficacy measures	X			X	X	X
Demographics	X					
SCID	X					
Internet Use and Access	X			X	X	X
Recovery Style Questionnaire	X			X	X	X
Peer Identification Scale	X			X	X	X
Psychosis Attachment Measure	X			X	X	X
WTAR	X					
Cognitive tests	X					
Engagement measures			X	X	X	X
Medication and psychological treatment	X			X	X	X
Expectancy Scale			X			

#### Primary outcome measure

##### Personal recovery

The primary outcome will be consumer-defined personal recovery, measured with the Process of Recovery Questionnaire (QPR [57]). This is a 22-item psychometrically validated self-report measure, developed collaboratively with mental health service consumers to measure recovery in people who experience psychosis [57]. Items are scored using a 5-point Likert scale. This measure was selected due to good alignment with the CHIME model on which the intervention is based [58]. The total score will be used as the main outcome measure, and the two subscales (for intrapersonal versus interpersonal recovery processes) will be reported.

#### Secondary outcome measures

##### Personal recovery dimensions

Two additional measures will be included for more focused measurement of specific personal recovery dimensions targeted by the intervention. Hope and optimism will be measured by the Schizophrenia Hope Scale, a validated 9-item self-report scale on which items are scored *disagree*, *agree* or *strongly agree* [59]. Social connectedness will be indexed by version 3 of the UCLA Loneliness Scale [60], the most widely used measure of loneliness versus connection with others, which comprises 20 items which are self-rated on a 4-point response scale from *never* to *always*. The total scores on each of the measures will be used in analyses.

##### Psychotic symptoms

Psychotic symptom severity will be measured by the PANSS [61]. The PANSS is a standardised interview-based measure of schizophrenia symptomatology widely used in treatment trials. It comprises seven items assessing positive symptoms, seven assessing negative symptoms and 16 items assessing general symptoms. The PANSS total score is used as part of the randomisation stratification, and the total and subscale scores will be analysed as secondary outcomes. Inter-rater reliability on the PANSS is checked every 3–6 months by research assistants co-rating face-to-face assessments, with recalibration of ratings occurring if necessary.

Although the PANSS has become a standard in psychosis treatment research, it has been criticised for providing a relatively indirect and insensitive index of the impact of psychosocial interventions on psychosis symptoms, where the impact of psychosis on distress and functioning is the key target, rather than symptom severity per se [8, 62]. Hence, the impact of psychotic symptoms will be measured by the Subjective Experience of Psychosis Scale (SEPS [63]). This is a psychometrically validated [63] questionnaire on which participants rate the current impact of psychotic symptoms on various aspects of distress, day-to-day functioning and subjective experience identified as important by mental health service users. The negative impact of psychosis is measured by the total of 29 5-point (*not at all* to *very much negative*) items. The SEPS is completed only by participants who have experienced psychotic symptoms in the week prior to the baseline assessment.

##### Emotional symptoms

Emotional symptoms will be measured using the 21-item version of the Depression Anxiety Stress Scale (DASS-21, [64]), which consists of 3 subscales (Depression, Anxiety, Stress) comprising items rated on a 4-point scale of the extent to which the experience occurred over the past week from 0 (*did not apply to me at all*) to 3 (*applied to me very much or most of the time*). The DASS-21 is validated and psychometrically robust [64].

##### Quality of life

Quality of life will be measured using the Assessment of Quality of Life (AQoL)-8D [65] consisting of 35 items across eight dimensions comprisingphysical (independent living, pain, senses) and psychosocial (mental health, happiness, coping, relationships, self-worth) dimensions of quality of life. The AQoL-8D is comprehensively validated and demonstrates high test-retest reliability [66]. The AQoL-8D total and physical and psychosocial scores will be used as a secondary outcome, and the utility scoring algorithm will be used to derive quality adjusted life years (QALYs) for an economic analysis which will integrate these data with those from other studies in the research program and be reported upon separately.

##### Collaboration with mental health services

We will also examine whether there are indirect effects of the intervention on the working relationship with the person’s usual services. This will be assessed using the Scale to Assess Therapeutic Relationships in Community Mental Health Care (STAR) Patient Version [67], a psychometrically validated self-report scale with 12 items on positive collaboration, positive clinician input, and non-supportive clinician input each rated from 0 (*never*) to 4 (*always*).

##### Subjective experience of intervention

At the three month assessment point, all participants complete questions asking “Overall, did the website make you feel better, or worse, or no different?” and “Do you feel that using the website made the impact of your mental health problems better, or worse, or no different?”, both answered on a 5-point scale ranging from *much worse* to *much better*. As well as providing an index of the subjective perceived benefit of the interventions, this measure will be used to assess for any perceived adverse effects. Although not a formally validated measure, analogous questions have proved sensitive to treatment condition in two previous trials of psychosis [55, 56]. In addition, a sample of SMART participants will be invited to take part in more detailed qualitative interviews about their experiences of the intervention, which will be reported upon separately.

##### Serious adverse events

In line with the Australian National Statement on Ethical Conduct in Human Research [68], in the context of this trial serious adverse events include events that lead to participant death, or that are life-threatening, require inpatient hospitalisation, or result in persistent or significant disability/incapacity. During the trial, potential serious adverse events are recorded, and are reviewed to determine whether they are likely to be related to interventions used in the trial. Analysis of outcomes will include a comparison of the number of participants in which an adverse event has occurred, and report of any adverse events attributed to either of the conditions.

##### Service use

Although differences in service use are not formally hypothesised as an outcome of the intervention, a resource use questionnaire (RUQ) is administered in order to assess for any differences in mental health service utilisation between the groups before, during, and following intervention. This will be used to derive costings for the economic analysis. The RUQ was modified for the local context of the study, assessing use of mental health services in Victoria including hospital admissions (number and days) and consultations with mental health service providers (number and length). This will be supplemented by Australian Government Medicare data: these administrative data are complete, but do not include the full spectrum of services which participants in this study are likely to use.

#### Process variables

##### Self-stigma

Self-stigma will be measured by the Internalised Stigma of Mental Illness Scale (ISMI), a psychometrically validated self-report measure with 29 4-point *strongly agree* to *strongly disagree* items which provide a total, and separate scores for Alienation, Stereotype Endorsement, Discrimination Experience, Social Withdrawal and Stigma Resistance [45].

##### Self-efficacy

Self-efficacy for personal recovery is measured by the item “How confident are you that in the future you will be able to live a satisfying life alongside any mental health problems you may have” rated from 0 (*not confident I can do this at all*) to 100 (*highly confident I can do this*). Also included, using the same response scale, are a self-efficacy for illness self-management item (“How confident are you that you can do things to manage any future mental health difficulties”), and a series of 12 items relating to content of the website. A 0–100 rating of confidence in achieving full clinical recovery is also administered to assess discrimination from this construct. To assist with validation of this new measure, the widely used Generalised Self-Efficacy Scale (GSES [69]) is also completed.

#### Covariates and moderating variables

Demographics collected include age, gender, employment, education, country of birth and ethnicity. Clinical variables include SCID diagnosis, age at first specialist mental health service contact, past psychiatric admissions, and past receipt of psychiatric treatment on an involuntary basis. Ratings are also obtained of level of independent use of the Internet and email (each rated as use without assistance, use with occasional assistance, use only with assistance); frequency of use of the Internet, email, and social media; and membership of mental health related forums or social networks. Additionally the following variables are collected:

##### Recovery style

Recovery style refers to individual differences in adaptive coping during recovery from psychosis, which range from an *integrative* style in which the person approaches their psychotic experiences with curiosity and interest, to a *sealing over* style in which they are encapsulate their psychosis and see it as separate [70]. Past studies have suggested that persons with a sealing over style benefit less from interventions that involve discussion of mental health [71] making this a likely moderator of outcome. This will be measured using the Recovery Style Questionnaire, a self-report measure of 39 true/false items with good psychometric properties [72].

##### Mental health peer contact and identification

Because the intervention is primarily based upon peer delivery, its effects may be influenced by the extensiveness and quality of prior peer contact, and the degree to which the person identifies with mental health consumer peers. Positive peer contact is assessed using a brief description of experiencing a positive recovery from psychosis, to which the person indicates how many people they have encountered fitting this description on a 4-point ordinal scale. Peer group identification is assessed on a scale adapted from that of Watson et al. [73]. Items, rated on a 9-point scale from *not at all* to *very much*, ask the extent to which the person identifies with, feels strong ties with, and sees themselves as part of the group of people that might be referred to as mental health consumers/patients/service users; how often they think about themselves as part of this group; and how close they feel to other members of the group. Higher scores indicate greater group identification. The original adaptation of this scale has good internal consistency [73].

##### Attachment

Because of the explicitly interpersonal nature of the intervention, both in the form of the face-to-face sessions, and the use of peer material, attachment style is included as a potential moderator. This is measured by the Psychosis Attachment Measure [74], a brief 16-item self-report questionnaire designed to assess avoidant and anxious attachment styles in persons with psychotic disorders.

##### Cognitive function

To determine whether cognitive function limits ability to benefit from resources, the WTAR will be used to control for premorbid IQ, in combination with tests of current cognitive functioning. The WTAR is administered as a test of premorbid IQ where participants are asked to read a list of 50 words that have atypical grapheme to phoneme translations (e.g., “liaison”, “ paradigm”) and are scored according to accuracy of pronunciation (raw score range 0–50). WTAR raw scores are standardised based on age then converted to the predicted WAIS-III IQ. While predicted IQ scores will be reported descriptively, these are associated with very large confidence intervals; standardised scores will therefore be used for analyses due to their greater precision. A small battery of cognitive functioning tests will additionally be administered. These include *Digit Symbol Coding* [76]*, Animal Fluency, Trails A and B* [76]*, Digits Forward and Digits Backward*. These tests assess the cognitive domains of executive function, verbal working memory, cognitive processing speed, task-switching and visual attention. Each assessment involves completion of paper and pencil cognitive assessments which are among the most frequently used in research and clinical practice and are well standardised with accepted norms. The battery takes a total of 8–10 min to complete. Each task will be briefly described below:

*Digit Symbol Coding* [75]*:* Participants are asked to use a key to write the numbers in a grid that correspond to nonsense symbols over a 90 s period. The total number of correct responses in used as the performance measure.

*Animal Fluency:* Participants are asked to give the names of as many animals as they can think of within a one-minute period. The number of unique animals produced is used as the primary performance measure, with repetitive errors or perseverations also noted.

*Trails A and B* [76]: For part A the participant is required to connect a series of 25 numbers in a circle as quickly as possible. They are asked to connect them in sequential order as quickly as possible without removing their pencil from the page. In part B the participant alternates between numbers and letters (i.e. 1, A, 2, B etc.). If the participant makes an error the experimenter corrects them before they move to the next circle. The time taken to complete each part is used as the performance metric.

*Digits Forward and Digits Backward:* For the forward condition participants are presented with lists of digits and must immediately repeat them. The length of the list increases on each trial. Participants are given two versions of each list length and score 1 point for each. After failing both versions of a list length the task is terminated. The total score is then calculated. For the backward condition, the participants need to reverse the order of the numbers.

Each of the primary variables for each task will be standardised based on age: Using these standardised scores a global mean current cognitive performance score will be calculated and used as a covariate in the analyses.

##### Intervention engagement/dose

Number of sessions attended and amount of time spent on each session will be recorded. In the SMART condition, access of the online materials using the participant’s username will be recorded automatically and usage within sessions and outside of sessions recorded.

##### Receipt of other treatments

Antipsychotic medication doses and receipt of psychological treatment will be tracked at each assessment point. These will be compared between groups, and to exclude these as accounting for outcomes, their relationship with outcome will be examined (medication doses converted into chlorpromazine equivalents).

### Data analysis

Data are initially recorded on paper forms which are securely stored and extracted into password protected electronic files. Data extraction is conducted by research staff blind to participant allocation. Procedures to identify potential errors include double entry from selected paper files, range checks, and examination of outliers.

The primary focus of the analysis is differential changes in the SMART group versus Befriending over the full post intervention period. Analyses will be undertaken using mixed-model repeated measures (MMRM) allowing for autoregressive dependence. MMRM is well-suited to ITT analyses because this approach uses all available information from subjects. Participants with incomplete data are not discarded and missing data are not replaced with estimated values or observations carried forward. Instead maximum-likelihood estimation is applied with the available data. Where there are significant group x time interactions, planned contrasts will compare changes from baseline under each intervention.

Where outcome measures exhibit non-normal distributions across time points, appropriate transformations will be applied. If any of the outcome measures show significant baseline differences between SMART and Befriending, and between completers and noncompleters, corrections for sample imbalance and attrition bias will be applied using propensity scoring. This will involve firstly constructing a logistic regression model with treatment condition as the outcome, and baseline clinical variables and demographics as predictors, plus a “missing-at-post-therapy” variable to index differential effects of attrition. Secondly the effects of any imbalance or attrition will be addressed by including the estimated probabilities from this model (propensity scores) as a covariate in analyses.

The differential effects on outcomes of the process variables (self-efficacy and self-stigma) and medication changes will be tested across conditions with a maximum likelihood Hierarchical Linear Modelling analysis. In these models time will be treated as a linear covariate. Baseline measures for cognitive function, recovery style, intervention engagement, diagnosis, demographics, baseline internet use and expectancy will be used as covariates, while testing interaction effects for condition and the baseline social engagement moderators (loneliness, attachment, peer group identification and peer contact). Structural equation modelling will then be used to demonstrate the mediation mechanism of outcome improvement for each condition separately, with bootstrap analyses used to obtain 95 % confidence intervals for all effect sizes.

Data will also be used as part of a broader economic evaluation of the use of the website involving cost-consequences analyses whereby incremental costs of interventions using the site are compared with the full spectrum of outcomes measured vs relevant comparators. For this study, QALYs will be calculated from AQoL8D data, and costs will be derived from estimates of the costs involved in running the website (website development and maintenance costs, forum moderation, and therapist activity data), adjusted for any differences in resource use between conditions. Standardised economic evaluation techniques will be used including incremental analysis of mean differences, with confidence intervals derived using bootstrapping.

## Discussion

This trial investigates the efficacy of a novel intervention based on putative personal recovery processes (CHIME) and common self-management materials. It will contribute a rigorously evaluated and disseminable intervention to the small but growing literature on mental health service interventions designed to support personal recovery from psychotic disorders. The trial has innovative elements including the integration of digital technology into mental health appointments using tablet computers, and the use of lived experience material to complement non-peer delivered mental health work. Examining this as a focused intervention in this study provides a potential model of low intensity provision in practice. As well as providing an intervention model for personal recovery in persisting psychosis, the trialling of using lived experience content as part of a non-peer intervention can also inform analogous interventions for different populations. Furthermore, examination of mechanisms of change associated with the lived experience content will be useful to inform both the use of this intervention, as well as informing the use of peer support more broadly.

## Abbreviations

AQoL-8D: Assessment of quality of life - 8 dimension
CHIME: Connectedness-hope-identity-meaning-empowerment
DASS-21: Depression anxiety stress scale 21
DSM-IV-TR: Diagnostic and statistical manual of mental disorders IV text revision
GSES: Generalised self-efficacy scale
HREC: Human research ethics committee
IQ: Intelligence quotient
ISMI: Internalised stigma of mental illness scale
ITT: Intention-to-treat
MMRM: Mixed-model repeated measures
PANSS: Positive and negative syndrome scale
QALY: Quality adjusted life years
QPR: Process of recovery questionnaire
RCT: Randomised Controlled Trial
RUQ: Resource use questionnaire
SCID: Structured clinical interview for DSM-IV-TR Axis I Disorders
SEPS: Subjective experience of psychosis scale
SMART: Self-management and recovery technology
STAR: Scale to assess therapeutic relationships in community mental health care
UCLA: University of California, Los Angeles
WAIS-III: Wechsler adult intelligence scale III
WTAR: Wechsler test of adult reading
